# Beyond movement: the dynamic roles of Type IV pili in cyanobacterial life

**DOI:** 10.1128/jb.00086-25

**Published:** 2025-07-03

**Authors:** Jonas Hammerl, Nils Schuergers, Gen Enomoto, Conrad W. Mullineaux, Annegret Wilde

**Affiliations:** 1 Molecular Genetics, Institute of Biology III, University of Freiburg 9174, Freiburg, Baden-Württemberg, Germany; 2 Spemann Graduate School of Biology and Medicine (SGBM), University of Freiburg 9174, Freiburg, Baden-Württemberg, Germany; 3 Department of Agricultural Chemistry, Tokyo University of Agriculture 13126, Setagaya, Tokyo, Japan; 4 School of Biological and Behavioural Sciences, Queen Mary University of London 4617, London, United Kingdom; Geisel School of Medicine at Dartmouth, Hanover, New Hampshire, USA

**Keywords:** Type IV pili, cyanobacteria, phototaxis, natural competence

## Abstract

Type IV pili are versatile prokaryotic cell appendages that are extremely widespread in the bacterial and archaeal domains of life. The structure, dynamics, and functions of type IV pili have been most intensively studied in several species of heterotrophic bacteria, but these appendages also appear universal in cyanobacteria. Cyanobacterial type IV pili have much in common with those found in other bacteria, but they also show some unique features that may be crucial for facilitating the photoautotrophic lifestyles of cyanobacteria. Here, we discuss what is known of the structure and dynamic organization of cyanobacterial type IV pili. We discuss the multiple roles of cyanobacterial type IV pili in motility and phototaxis, sensory signal transduction, DNA uptake, and the formation of cell aggregates and biofilms. We conclude with some ideas on the likely importance of cyanobacterial type IV pilus functions in the natural environment and for biotechnological applications.

## INTRODUCTION

The advent of oxygenic photosynthesis by ancient cyanobacteria approximately 2.4 billion years ago profoundly transformed Earth’s landscape. Today, cyanobacteria inhabit a vast array of aquatic habitats and less obvious places such as symbiotic associations, Antarctic rocks, and desert soil crusts, where they play pivotal roles in global nutrient cycling (1–3). Cyanobacteria are morphologically diverse, with well-studied model organisms including the single-celled *Synechocystis* sp. PCC 6803 (hereafter *Synechocystis*), the first phototrophic organism to have its genome sequenced in 1996 (4), as well as filament-forming, multicellular, and diazotrophic strains such as *Nostoc punctiforme*.

Many cyanobacteria inhabit surface-associated communities, where they interact with their surroundings, aggregate, migrate to favorable environments, adapt their colony architecture, or seek out symbiotic hosts. These behaviors rely to a significant extent on type IVa pili (T4P), which were first identified by homology with the type IVa pili of *Myxococcus xanthus* and *Pseudomonas aeruginosa* (5, 6) and are the only type IV filament superfamily systems encoded by cyanobacteria (7). T4P are dynamic surface filaments that undergo repeated cycles of extension and retraction. The T4P apparatus is a sophisticated, multi-protein machinery that is employed by a vast variety of bacteria and archaea and is closely related to the type II secretion system (T2SS) and the archaellum (8–10). T4P play vital roles in cell-environment interactions, including cell movement via twitching motility on solid surfaces, cell-cell interactions, DNA uptake, and biofilm formation.

There were early observations of pilus-like appendages on the surface of a number of unicellular and filamentous cyanobacterial strains (11, 12). Genomics and subsequent mutational studies in multiple cyanobacterial species revealed the presence of T4P genes that are required for many typical T4P functions, including pilus assembly, surface motility, natural competence, and cellular aggregation (5, 6, 13–18). Apart from T4P, other filaments have been observed in electron micrographs of cyanobacterial cells. So-called thin pili with an average diameter of 3–4 nm and a length of approximately 1 µm cover the entire cell surface of *Synechocystis*, where they can form bundles. While some mutants defective in T4P assembly seem to lack thin pili, they are clearly different from T4P as their biogenesis does not depend on the major pilin PilA1 or a functional T4P machinery (5, 6). To date, their structure and function remain unknown.

The functions of T4P, along with their regulation and ecological implications, are as diverse as cyanobacteria and their ecological niches. Despite their ecological significance, T4P-dependent behavioral responses in cyanobacteria remain underexplored, leaving substantial gaps in our understanding of cyanobacterial T4P. However, the conservation of T4P components in most cyanobacteria indicates that both unicellular and filamentous strains share a common T4P architecture that performs its various functions based on the same principles, such as repeated cycles of polar pilus assembly and retraction (7, 15, 17, 19, 20). This review aims to summarize the current state of research on the various functions of the cyanobacterial T4P machinery.

## THE CYANOBACTERIAL T4P

**Fig 1:**
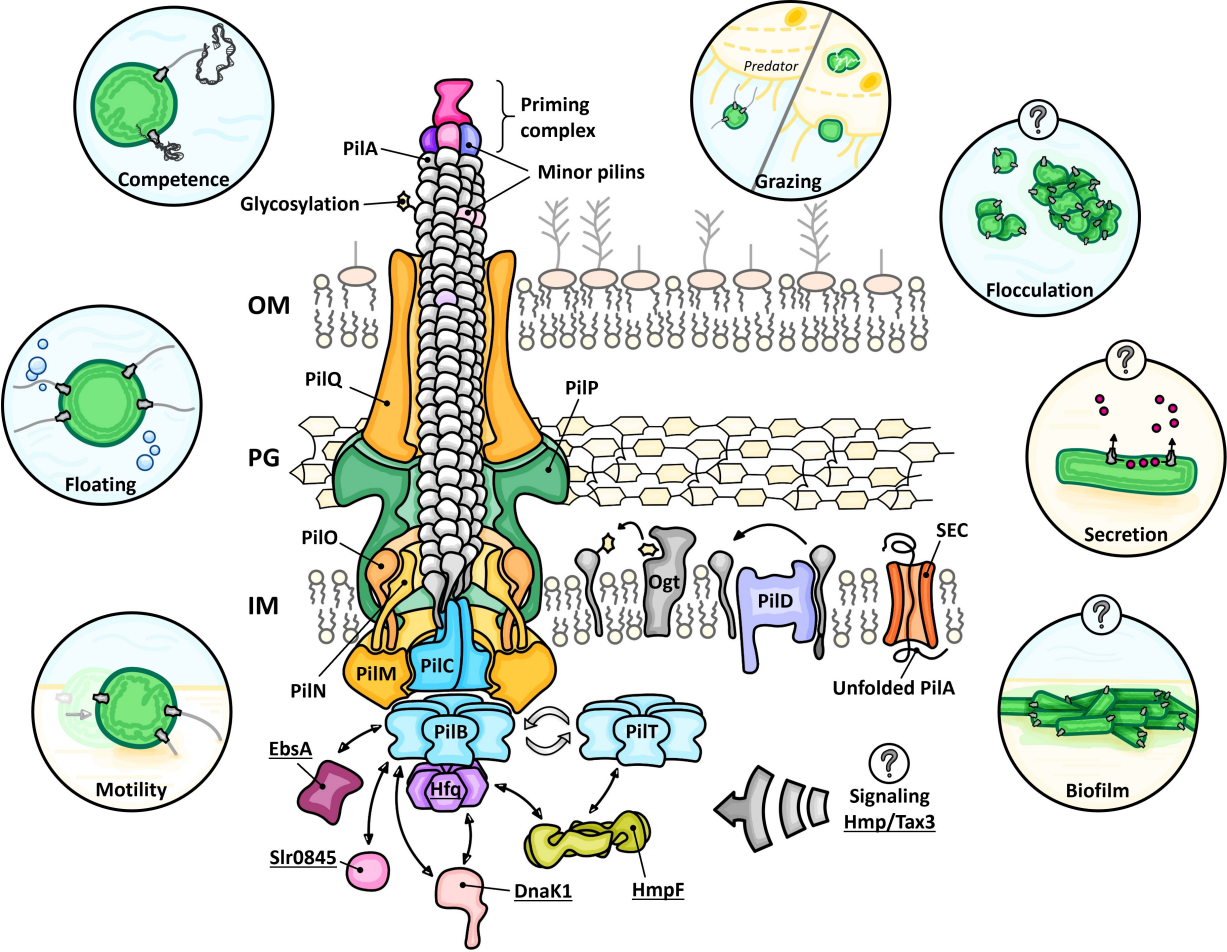
Structure of the cyanobacterial type IVa pilus (T4P). The basal complex of the T4P spans the cell envelope via the outer membrane secretin PilQ, PilP, and the inner membrane proteins PilMNO. PilA pilin precursors are exported in an SEC pathway-dependent manner and inserted into the inner membrane. Pilins are processed by PilD and an Ogt enzyme. Different minor pilins can assemble the priming complex necessary for T4P polymerization or be integrated into the filament directly. Pilins are inserted into or removed from the pilus fiber by conformational changes of the platform protein PilC mediated by the antagonistic motor ATPases PilB and PilT. T4P activity is regulated at the pilus base, with cyanobacteria-specific factors such as Hfq, EbsA, and HmpF playing critical roles for T4P assembly. The exact role of T4P in flocculation, biofilm formation, and secretion of substrates other than T4P fibers remains unclear. Factors specific for the cyanobacterial T4P are underlined. The surface (S-)layer present in most cyanobacteria, but absent in the motile *Synechocystis* substrain (27), is not displayed as it is unknown how T4P traverse or interact with the S-layer. Figure is not drawn to scale. OM, outer membrane; PG, peptidoglycan; and IM, inner membrane.

Cyanobacterial T4P proteins have not been investigated on a biochemical and structural level in detail. Nonetheless, their role in pilus assembly can be deduced from mutant phenotypes and their homology to the extensively studied T4P machinery in Gram-negative (diderm) model organisms (reviewed in reference 21), whose architecture in heterotrophic bacteria has been revealed through cryo-electron tomography (22–24). The pilus is anchored by a complex basal body embedded in the cell envelope (Fig. 1). This basal body consists of an outer membrane secretin pore (PilQ), which facilitates the transport of the pilus filament across the outer membrane, while an inner membrane platform protein (PilC) transduces force generated by ATP hydrolysis into pilus (dis-)assembly. Corresponding cytoplasmic motor ATPases interact with the platform protein to power pilus assembly (PilB) or disassembly (PilT). An alignment complex (PilMNOP) spans the periplasm and inner membrane to connect the secretin with the platform protein and motor ATPases. In nearly all cyanobacteria, the secretin and most alignment complex proteins are encoded within a conserved *pilMNOQ* gene cluster that, in contrast to the genomic organization in many other bacteria, does not encode a homolog of the lipoprotein PilP, which connects the inner and outer membrane components of the T4P machinery in diderms (20). Disruption of these genes leads to the loss of T4P and their functions (6, 25). Although it was not identified until recently, now there is evidence that cyanobacteria encode a PilP homolog independent of the *pilMNOQ* locus (17). Cyanobacterial PilP proteins generally seem to be longer than their homologs in *Myxococcus xanthus* and *Pseudomonas aeruginosa*, which might be a cyanobacterial adaptation to the thicker peptidoglycan layer compared to other diderm bacteria (26). However, if the genomic uncoupling of *pilP* and the alignment complex genes has functional or regulatory relevance, or is the result of genetic drift, remains enigmatic.

Little is known about the composition of the cyanobacterial T4P filament whose main constituent is the major pilin protein PilA. In addition, most (and perhaps all) cyanobacteria encode so-called minor pilins that are present in lower abundance. From heterotrophic model bacteria, we know that a conserved set of core minor pilins forms a tip-located priming complex, which is essential for initiating pilus assembly and plays a crucial role in pilus function. Other, less conserved, minor pilins can be distributed along the length of the filaments in specific T4P systems (reviewed in references 28, 29). The low sequence conservation of pilin genes complicates their categorization and comparison between different species. Nonetheless, mutational studies suggest that specific sets of minor pilins define the functional properties of the T4P filament and thus enable the same basic structure to be used for very different purposes. The implications of specific cyanobacterial minor pilins in different T4P functions will be discussed in the subsequent sections.

Pilin monomers, produced as prepilins with an N-terminal signal sequence, are inserted into the inner membrane via conserved hydrophobic amino-terminal α-helices. During maturation, the prepilin peptidase PilD cleaves the leader peptide and methylates the N-terminal amino acid of the mature protein (30). In cyanobacteria, inactivation of *pilD* leads to defective pilus assembly and induces the accumulation of unprocessed pilins in the thylakoid membranes where they interfere with photosystem assembly, likely by jamming SecY/YidC translocons (6, 14, 31, 32). Additional post-translational modifications of pilin subunits are thought to be critical for the biogenesis of functional pili. In many cyanobacteria, a gene encoding an *O*-linked β-N-acetylglucosamine transferase (Ogt) is located adjacent to the major pilin gene *pilA*, and it has been shown that Ogt is involved in PilA glycosylation and affects pilus assembly (33, 34). Protein glycosylation involves numerous enzymes, so it is not surprising that many genes have been identified that, directly or indirectly, cause the accumulation of pilins with abnormal sizes due to defects in glycosylation or other post-translational modifications (35–39). Furthermore, it has been suggested that trimethylation at the C-terminal lysine of PilA1 is essential for proper pilus formation and function in *Synechocystis* (40).

The dynamic nature of T4P is characterized by cycles of rapid extension and retraction, driven by the activity of two specific, mutually exclusive, motor ATPases. PilB facilitates the polymerization of pilin subunits into the growing pilus filament, while PilT mediates its depolymerization. Binding and hydrolysis of ATP by the hexameric motors lead to a conformational change of the platform protein PilC that is thought to trigger the integration or release of a pilin subunit from the pilus fiber (21, 41). Cyanobacteria typically encode a copy of each motor ATPase together with the pilus platform protein PilC in a conserved *pilBTC* gene locus. Disruption of *pilB1* or *pilC* abrogates pilus assembly, while disruption of *pilT1* leads to hyperpiliation causing a loss of T4P function (5, 6, 14, 25, 42). Furthermore, many strains encode a second PilT homolog (PilT2) characterized by a proline-rich N-terminus (20). Mutant strains of *pilT2* retain T4P on the cell surface (5, 14) and do not exhibit altered transformation efficiency (17). Bhaya et al. (5) observed that a *Synechocystis pilT2* mutant exhibited negative phototaxis although a plausible explanation for this phenotype is lacking. As these experiments were performed on only a single clone without complementation experiments, it is plausible to attribute the unexpected *pilT2* phenotype to secondary mutations. From our experience with *Synechocystis* motility and phototaxis, we know that under laboratory conditions, spontaneous mutations impacting T4P functions frequently arise and lead to loss of motility or, more seldom, negative phototaxis or aberrant biofilm formation. It is tempting to speculate that PilT2 acts similarly to PilU as a PilT-dependent retraction ATPase that is necessary for T4P retraction under load (43, 44). Albeit not as frequent as PilT2, additional homologs of the motor ATPases, like a second PilB homolog (20), can be found in a variety of different cyanobacteria, but the function of these additional homologs has not been elucidated.

## THE CYANOBACTERIAL T4P MOTOR AS A PROTEIN INTERACTION HUB

Recent research indicates that the assembly ATPase PilB is an interaction hub for unique cyanobacterial proteins that are essential for the assembly of functional T4P (Fig. 1). A remarkable example is Hfq, which is widely conserved as an RNA chaperone in other bacteria but unlikely to bind RNA in cyanobacteria (19). A conserved C-terminal extension of PilB mediates the interaction with Hfq, which localizes to the membrane in a T4P-dependent manner and is essential for pilus assembly by an unknown mechanism (19, 45–47). EbsA is a small, highly conserved protein only found in the cyanobacterial clade. It directly interacts with PilB and can be co-purified with PilB and Hfq, and similar to these proteins, it is crucial for pilus assembly (13, 47–49). Homologs of the *Synechocystis* Slr0845 protein—exclusive to clade B cyanobacteria, which comprise the majority of terrestrial and freshwater species (50)—are predominantly encoded immediately downstream of the *pilBTC* locus and interact with PilB and/or Hfq, albeit inactivation of *slr0845* does not affect pilus assembly (20, 47). Furthermore, DnaK1, along with a tetratricopeptide repeat (TPR) domain-containing DnaJ homolog (DnaJ3 in *Synechocystis* and *N. punctiforme*) and GrpE, comprise a chaperone system that specifically interacts with the T4P motor components PilB and Hfq. Reminiscent of the aforementioned *slr0845* homologs, cyanobacterial *grpE* and *dnaK1* genes are frequently located in the direct genomic vicinity upstream of the *pilBTC* gene cluster. While the DnaK1 protein machinery is not crucial for T4P assembly, it affects motility and is thought to link T4P activity to the production or secretion of extracellular polysaccharides (13, 51, 52).

HmpF is an important regulator of T4P function that interacts with Hfq as well as the retraction motor PilT (46). Homologous proteins, found exclusively in cyanobacteria, are often encoded adjacent to chemotaxis-like systems which are known to affect pilus assembly. In *N. punctiforme*, the chemotaxis-like system *hmp* regulates the dynamic localization of HmpF (37, 46, 53). HmpF shows sequence similarity with the coiled-coil region of Structural Maintenance of Chromosomes (SMC) proteins, and while nothing is known about its function, *hmpF* mutants are immotile and do not assemble T4P on the cell surface (13, 46, 53, 54).

While existing evidence convincingly suggests that the tripartite complex involving PilB, Hfq, and EbsA, along with the dynamic localization of HmpF, is essential for T4P assembly, the specific functions of these proteins remain unclear. We hypothesize that the interactions of these additional proteins with the motor ATPases are critical for facilitating efficient phototactic orientation in response to incident light, underscoring the specialized adaptation of the T4P system in cyanobacteria. Proteins associated with the pilus base are likely crucial for transmitting sensory information from the T4P to control gene expression. Consistent with this idea, mis-localization of Hfq in the absence of its PilB interaction partner results in changes in gene expression (19). Undoubtedly, the dynamic protein interactions at the pilus base are among the most crucial aspects of the cyanobacterial T4P machinery that warrant further investigation.

## T4P FUNCTION IN CYANOBACTERIAL MOTILITY AND PHOTOTAXIS

Cyanobacteria possess the ability to move directionally in response to environmental cues. Remarkably, cyanobacteria can directly sense light directions and optimize photosynthetic conditions by moving away from harmful or toward beneficial illumination in processes termed negative and positive phototaxis, respectively (55–57). Additionally, other responses to light, such as photophobic behaviors, have been described and are discussed in detail elsewhere (58).

In the past, different mechanisms such as slime secretion (59) or waves propagated by an array of surface fibrils (60, 61) have been proposed to power surface motility of cyanobacteria. However, many studies have provided evidence for broad conservation of T4P components in the cyanobacterial clade (15, 18), and T4P-mediated motility also in multiple filamentous strains (15, 25, 53, 62), supporting the hypothesis that most cyanobacteria employ T4P for surface motility. During T4P-mediated surface motility, referred to as twitching motility, T4P filaments are thought to act as molecular grappling hooks that extend, attach to a substratum (or a neighboring cell), and pull the cell forward during retraction (53, 63–65). These cycles of extension and retraction are powered by the alternating activity of the T4P motor ATPases PilB and PilT, providing the force that is necessary to pull the cell forward (66–68). Therefore, an overarching principle in T4P-mediated motility is the selective, polar activation of T4P motor complexes to enable directional movement.

**Fig 2:**
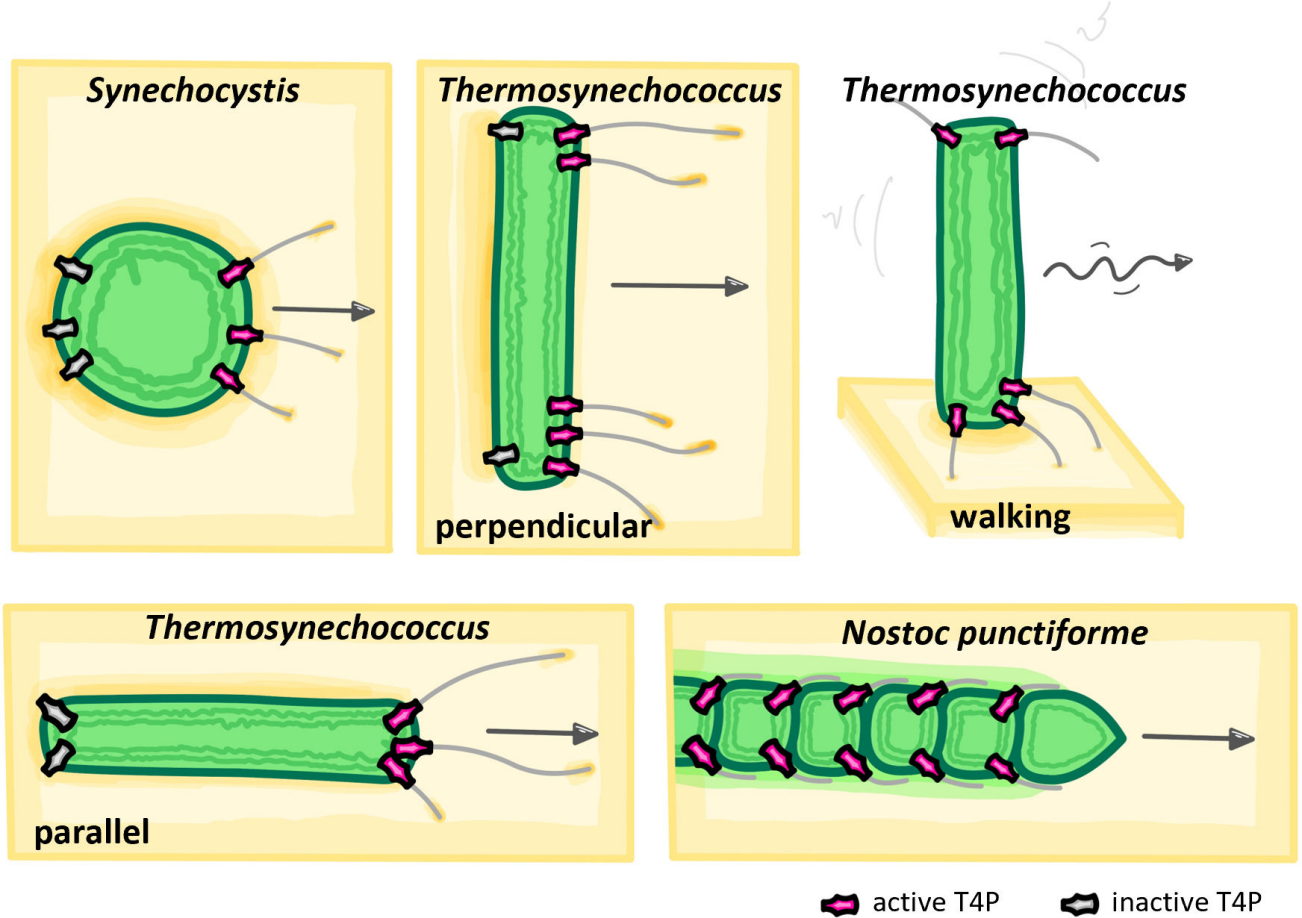
Cyanobacterial movement modes. Spherical *Synechocystis* cells move through the activation of T4P selectively on one side of the cell body, facilitated by the polar localization of PilB. In most cases, cells do not roll during movement (55). Rod-shaped cyanobacteria, exemplified by *Thermosynechococcus* species, can move parallel or perpendicular to their own long axis by activating T4P asymmetrically at either one pole or both cell poles simultaneously. Additionally, walking on one cell pole has been described. Filamentous cyanobacteria, such as *N. punctiforme* hormogonia, presumably move by cycles of polar pilus extensions and retractions. Pili adhere closely to the filament and are proposed to attach to secreted hormogonium polysaccharide.

To date, most research on the function of T4P and their regulation during motility and phototaxis has been performed in only a few model organisms: unicellular *Synechocystis,* rod-shaped *Thermosynechococcus* and *Synechococcus* species, and the filamentous *N. punctiforme* that can develop motile hormogonia, which serve as dispersal units (69). T4P are believed to be uniformly distributed around spherical *Synechocystis* cell bodies, and unidirectional activity of T4P is achieved by dynamic re-localization of PilB in crescents toward the direction of movement (55, 64, 70). Conceptually, polar switching of the motor ATPases PilB or PilT is a common way to control T4P activity in heterotrophic model organisms (71, 72), and might also be conserved in unicellular cyanobacteria. Rod-shaped *Thermosynechococcus vulcanus* cells are inherently bipolar with T4P emanating predominantly at the cell poles. Dynamic motor re-localization has been hypothesized but not demonstrated so far (63). Similar to *Pseudomonas aeruginosa* or *Myxococcus xanthus*, rod-shaped cyanobacteria can display movement along their longitudinal axis with active T4P only at one pole but are also capable of walking upright on a single pole, which confers less directional persistence (63, 73, 74). Interestingly, recent studies additionally reported continuous movement perpendicular to the long axis of cells during which pili are asymmetrically extended from both poles simultaneously (Fig. 2). This unique mode of movement displayed by *Thermosynechococcus vulcanus* (63) and a motile *Synechococcus elongatus* PCC 7942 strain (74) is distinct from heterotrophic model organisms (73, 75, 76) and requires that the asymmetric activation of the T4P machinery can be precisely regulated within a single cell pole (63) (Fig. 2). In contrast to single-celled strains, T4P motor complexes in motile hormogonia of *N. punctiforme* remain statically located in two bipolar rings close to the septa of individual cells within a filament. T4P are extended from these rings asymmetrically at the leading pole to move along their long axis (Fig. 2). In contrast to the static T4P motor ATPases, the coiled-coil protein HmpF shows dynamic re-localization to the leading pole during movement reversals. The polar localization of HmpF to the piliated pole is controlled by the Hmp chemotaxis-like system, likely upon perception of an unknown stimulus, perhaps proton motive force (46). Deletion of *pilT* reduced the frequency of HmpF re-localization, whereas deletion of *hmpF* abrogates the polar localization of the DnaK1 chaperone system, which interacts with PilB. These data suggest that HmpF might play a key role in regulating the polar activity of T4P motor ATPases to control directional movement in *N. punctiforme* (46, 48, 52, 53).

*Synechocystis* exhibits phototaxis in response to a wide spectrum of directional light stimuli (57, 77). Spherical *Synechocystis* cells act as biological microlenses, focusing incoming light onto the rear side of the cell, thereby creating a roughly fourfold intensity gradient across the cell body (55). This light gradient is transduced via an unknown signaling pathway to selectively activate T4P on the leading side of the cell via recruitment of PilB (55, 70). Although most work has been done in coccoid *Synechocystis* cells, there is increasing evidence that also rod-shaped cyanobacteria are capable of lensing (63, 78). The latter focus light in a focal point in each pole, creating a “pole-in-pole” during illumination parallel to the long axis, or along the entire light-averted side in illumination lateral to the long axis (63, 78). By contrast, the mechanisms of directional light perception in filamentous cyanobacteria remain largely unexplored. Interestingly though, a direct reaction to directional light stimuli has been observed in certain strains (79), while others enable phototaxis, at least partially, via reversals of movement along the filament axis in a biased random walk type of movement (53, 80). This might hint at different modes of signal integration, with single cells in a filament either being capable of resolving light direction spatially, as proposed in lensing, or sensing light intensity changes temporally.

In filamentous cyanobacteria, the *hps* gene cluster encoding a Wzx/Wzy-type polysaccharide synthesis and export system along with minor pilin genes is widely conserved (15, 81). It remains unclear whether hormogonium polysaccharide (HPS) is secreted through the PilQ outer membrane pore by cycles of pilus extension and retraction, as initially hypothesized, or if HPS is exported by a later identified Wza-type polysaccharide export protein (15, 81, 82). Irrespective of its export route, HPS is essential for hormogonium motility. However, contrary to earlier hypotheses, its secretion does not generate the propulsion force for cell motility, as exogenous addition of conditioned medium complements the *hps* phenotype (15). In *N. punctiforme* hormogonia, HPS likely provides a surface for T4P attachment during gliding motility, similar to S-motility in *Myxococcus xanthus* (53, 83). Although the responsible genes remain unidentified, exopolysaccharide (EPS)/slime secretion likely aids *Synechocystis* motility by reducing surface friction (84), despite the exact chemical composition of *Synechocystis* EPS being distinct from HPS in filamentous strains (81).

Though we lack a detailed picture of phototactic signal transduction, chemotaxis-like systems containing two response regulators are known as central T4P regulators. Systems classified as type 1 (85), like *N. punctiforme hmp* (37, 82) or *Synechocystis tax3 (*86), seem to generally control proper, coordinated T4P assembly and likely act via HmpF (46, 53). Moreover, systems comprising photoreceptor modules control phototactic orientation in *Synechococcus elongatus* UTEX 3055 (78, 87), *Synechocystis* (88, 89), and *N. punctiforme* (90). For *Synechocystis,* the phosphorylated response regulator PixG, which contains a PatA N-terminal (PATAN) domain (89, 91), interacts with PilC and PilB1 and localizes to the leading cell pole under directional light, presumably promoting positive phototaxis via polar recruitment or activation of PilB. The CheY-like regulator PixH acts antagonistically and inhibits positive phototaxis (88). Additional PATAN regulators also interact with PilB1 but induce negative phototaxis (92–95).

In spite of substantial knowledge about downstream signal transduction, the question of how light direction is sensed upstream remains enigmatic. Considering the spectral overlap between phototaxis and photosynthesis action spectra in the blue and red, photosystems or the photosynthetic electron transport chain may well be involved in the resolution of light direction (96). Signal transduction from the photosynthetic complexes to T4P would likely have to occur via diffusible signaling molecules, given that photosystems are localized in the thylakoid membrane system and thus spatially separated from the T4P in the inner membrane (97, 98). Further research is essential to uncover how photoreceptors, potentially including the photosynthetic apparatus, along with HmpF and chemotaxis-like systems, collectively form a dynamically interconnected signal transduction network to establish polarity during cyanobacterial phototaxis.

## DNA UPTAKE

The ability of bacteria to take up DNA from their surroundings is known as natural competence. Imported DNA is not only used for processes involving homologous recombination, such as the repair of damaged DNA or the integration of foreign DNA into the genome to increase genetic diversity. DNA can also be used as a nutrient in scarce environments (99, 100). Both diderm and monoderm (Gram-positive) bacteria utilize T4P or related competence pili for DNA uptake. Despite significant research efforts, the detailed molecular mechanisms of T4P-mediated DNA transport are not fully understood. The current model (reviewed in reference 101) suggests that DNA binds to the pilus and is transported into the cell through pilus retraction, which moves the DNA through the outer membrane and peptidoglycan layer via the PilQ secretin channel. Once in the periplasm, DNA is bound by ComEA, acting as a Brownian ratchet, to prevent it from diffusing back. The DNA is then converted to single-stranded DNA (ssDNA) for transport across the inner membrane by ComEC. In the cytoplasm, ssDNA is further processed and can be incorporated into the genome through homologous recombination. Minor pilins, which act as DNA receptors, play a crucial role in the initial steps of this process (102–105) and presumably bind the DNA at the pilus tip, highlighting the role of the tip-located priming complex in natural transformation (106–108).

Natural competence in cyanobacteria was first identified in 1970 in the strain *Synechococcus elongatus* PCC 7942 (*Anacystis nidulans* 602) (109). Subsequent studies demonstrated the feasibility of genetic engineering via natural competence in important unicellular model cyanobacteria, including *Synechocystis* (110), *Synechococcus* sp. PCC 7002 (111), and *Thermosynechococcus elongatus* (112). T4P-dependent natural competence has also been observed in filamentous and branched strains, such as *Phormidium lacuna* and *Chlorogloeopsis fritschii* PCC 6912 (16, 113). These strains possess genes encoding the fundamental T4P machinery and competence proteins essential for DNA internalization. Genomic analyses suggest that at least 60–70% of cyanobacterial species, including those from the early-diverging *Gloeobacter* lineage, have a complete set of genes predicted to be crucial for the uptake of exogenous DNA (113–116). Consequently, natural competence is likely an ancient trait in cyanobacteria that was lost during evolution in some lineages like marine, free-living picocyanobacteria but may be more frequent than previously reported.

Direct observation of DNA binding and uptake by T4P in cyanobacteria is lacking; however, mutational analysis in *Synechocystis* and *Synechococcus elongatus* PCC 7942 has confirmed that nearly all genes encoding the core T4P machinery and competence proteins ComA/E/F and the ssDNA-binding DprA are required for DNA uptake (5, 6, 17, 42, 117). Additionally, the proteins EbsA and Hfq, forming a cyanobacteria-specific tripartite complex with PilB to control pilus assembly, are crucial for natural transformation (45, 47). Mutation of the *pilB2* gene in *Synechocystis* leads to reduced transformation efficiency, whereas data from *Synechococcus elongatus* PCC 7942 indicate that inactivation of retraction ATPases other than PilT1 does not impact transformation efficiency (6, 17).

As in other bacteria, minor pilins play a significant role in DNA uptake. In *Synechocystis*, the minor pilin *pilA5*, but not the co-transcribed pilin *pilA6*, is essential for transformation but not for motility or other T4P functions (118). Similarly, the inactivation of PilA2, which is homologous to the major pilin PilA1, does not affect motility but reduces transformation efficiency (6). In *Synechococcus elongatus* PCC 7942, the minor pilins PilA3, PilW, and the putative pilins RntB and RntA are required for natural transformation (17). Due to the generally low sequence identity of pilins, the homology between different pilins is difficult to define. Hence, it is challenging to determine which minor pilins are generally conserved in cyanobacteria and which may constitute a putative tip complex mediating DNA uptake. In *Synechocystis*, blue light stimulates the diguanylate cyclase activity of the cyanobacterial phytochrome Cph2, leading to increased cellular levels of c-di-GMP (119, 120). This leads to the repression of the *pilA5-pilA6* operon and the minor pilin *pilX1* (121). Furthermore, the expression of *pilA5-pilA6* and *pilX1* is upregulated upon surface contact on agar plates compared to planktonic cultures. Inversely, the *pilA9-pilA12* mRNA is more abundant in planktonic cultures and under blue light (118, 121). Based on these data, we hypothesize that, depending on environmental conditions, *Synechocystis* cells express different sets of minor pilins that form different tip complexes. Minor pilins, which are involved in natural competence, may bind DNA, as has been shown in *Neisseria* (102). It seems sensible that DNA uptake is enhanced in environments with high cell densities such as biofilms or densely growing agar plates. Similarly, due to chlorophyll absorption, higher cell densities result in a green light-enriched environment. Therefore, a high blue-to-green light ratio might be used as an indicator of low-density planktonic conditions in which DNA uptake functions should be repressed (122).

## FLOCCULATION AND BIOFILM FORMATION

Cyanobacteria frequently aggregate and form biofilms both in laboratory settings and in the wild, where they are associated with other bacteria. Within a biofilm, cells are linked to each other and embedded into a matrix of extracellular polymeric substances. The multicellular bacterial communities can be surface-attached, as seen in microbial mats, or non-attached, like those aggregates or flocs often found in cyanobacterial blooms. Multiple physiological benefits have been proposed for these assemblages, including flotation in water columns, self-shading from harmful light, building symbiotic microbial communities, and defense from predation (123, 124).

T4P play a role in surface attachment and biofilm formation in bacteria and archaea (reviewed in references 125–127). However, their role in cyanobacterial aggregation and biofilm formation is not well understood, and a limited number of studies across different species do not present a consistent picture. Research on *Synechocystis* flocculation (the aggregation of suspended cells into large floating clusters) provided evidence for the essential involvement of T4P in this process. Mutation of different genes belonging to the chemotaxis-like *tax3* operon, which is important for pilus biogenesis, promoted autoaggregation (128). Inactivation of the T4P machinery led to mutants devoid of T4P that did not flocculate (129, 130). Moreover, the minor pilins PilA9-12, which are important for T4P surface motility and are expressed under high intracellular c-di-GMP concentrations, are important for the flocculation process. It is suggested that one or more of these minor pilins mediate a specific adhesion process strictly required for floc formation (118, 130). Interestingly, the dynamics of pilus retraction appear to be less critical since mutants lacking the retraction motor PilT1 still form flocs. These observations suggest that T4P primarily facilitate the initial adhesion of cells rather than actively pulling them together. Apart from T4P, the export of a specific sulfated EPS, called synechan, has been shown to be essential for *Synechocystis* flocculation (131). Similarly, PilB and presumably assembled T4P are important for microcolony formation in *Thermosynechococcus vulcanus* (63). Observed macroscopic flocculation in this strain depends on the export of cellulose rather than synechan (132), but the requirement for T4P in this process has not been studied. However, it was observed that coaggregation between the closely related strain *Thermosynechococcus* sp. NK55a and *Chloroflexus aggregans* depends on pilus assembly and is severely diminished in a *pilB* mutant (133). Likewise, deletion of both *ebsA* and *pilB* abolished biofilm formation in *N. punctiforme* (48). In contrast, biofilm formation in *Synechococcus elongatus* PCC 7942, which is by default suppressed in the laboratory (134), does not require T4P (135). On the contrary, mutations that abrogate T4P assembly relieve suppression and enable biofilm formation through amyloid fibrils formed by proteins encoded in the EbfG operon (135–137). This indicates variability in the necessity of T4P for aggregation across different cyanobacterial species, underscoring the complexity and species-specific nature of biofilm formation mechanisms.

We lack a detailed, step-by-step picture of *Synechocystis* floc formation, and consequently, the reasons for the requirement for both T4P and synechan production are not clear. One possibility is a link between pilus activity and EPS production and export similar to *N. punctiforme*, where motility is linked to HPS production (see above). In *N. punctiforme,* mutation of either *pilB* or *pilQ* abolishes EPS production (15). However, despite the strong homology of T4P with the T2SS, direct involvement of *Synechocystis* T4P in secretion has never been demonstrated (138). EPS production may be closely associated with the activity of the T4P systems via the DnaK1 chaperone system, which comprises DnaK1, DnaJ, and GrpE. This chaperone system interacts with the T4P motor ATPase PilB1 and affects EPS accumulation through an as-yet-unknown mechanism (52). A regulatory connection between T4P-mediated sensing of the physical environment and EPS production is very plausible in *Synechocystis* (118). A second, not mutually exclusive, idea would be that flocs are assembled by the adhesion of cells to strands of EPS, and that T4P are needed for effective adhesion (124). This would be consistent with the rather passive role of T4P that is implied by the strong flocculation of a mutant lacking the PilT1 pilus retraction motor (130).

## OUTLOOK ON ECOSYSTEMS AND BIOTECHNOLOGY

Cyanobacteria, among Earth’s most ancient and abundant organisms, play critical roles in various ecological systems. T4P-mediated behaviors in cyanobacteria have profound ecological implications, influencing their survival, growth, and interactions within diverse environments.

Cyanobacteria grow either as freely suspended planktonic cells, floating aggregates, or attached to surfaces. In open-water bodies, marine *Synechococcus* and *Prochlorococcus* species utilize T4P to increase drag, thus remaining suspended at the optimal depth in the water column, while T4P simultaneously helps to evade grazing by bacterivores (18). *Trichodesmium*, a nitrogen-fixing cyanobacterium, often forms colonial aggregates in response to stress. These millimeter-sized colonies create unique microenvironments, performing different ecological and metabolic functions compared to single filaments (139). T4P-based gliding motility at the single filament level is proposed to be a crucial factor for the aggregation behavior of *Trichodesmium* and ultimately for its biogeochemical role in the ocean (140).

In sediments, soils, and habitats at phase boundaries, cyanobacteria attach to surfaces, often forming elaborate biofilm structures. In these environments, T4P-based motility is vital for dispersal and phototaxis, such as the collective behavior in *Synechocystis*, which forms finger-like clusters, allowing migration toward optimal light environments (5, 57). Chemokinetic and chemophobic motility responses are vital for the spatial organization of the cyanobacterial biocrust pioneer *Microcoleus vaginatus* (141). In other strains, the development of motile hormogonia is a prerequisite for establishing nitrogen-fixing symbioses with eukaryotic partners, such as the hornwort *Anthoceros punctatus* and the water fern *Azolla* (142, 143). Complex morphological patterns, like reticulate structures, arise from the undirected gliding and colliding of filamentous cyanobacteria. These self-organized structures can template more complex supracellular architectures, providing rigidity and enabling collective mechanical responses to external cues (144–146). This behavior is critical for the formation of microbial mats where cyanobacteria create microhabitats for diverse microbial communities. Stromatolites represent such ecosystems, which date back approximately 3.5 billion years (147).

The concept of emerging properties in multispecies microbial communities is growing. The T4P-dependent aggregation and phototactic motility of thermophilic cyanobacteria are greatly bolstered by co-cultivation with *Chloroflexus*, the co-habitant at hot springs (133, 148). These findings underpin the importance of physiological characterization in the laboratory in the context of ecological settings.

Interestingly, T4P-dependent phototaxis of *Synechocystis* is greatly enhanced by ethylene, though it produces a minimal amount of ethylene itself (149). Ethylene is a central plant hormone, regulating various processes during plant development (150). Putative ethylene sensors are abundant in filamentous cyanobacteria capable of nitrogen fixation and plant symbiosis, suggesting that ethylene-based regulation of T4P is a critical adaptation of cyanobacteria to symbiotic interactions with plants (151).

From a biotechnological perspective, recent advancements have highlighted the application of cyanobacterial biofilms as whole-cell biocatalysts, offering a robust alternative to suspension cultures (reviewed in reference 152). During production workflows, biofilms can be exploited in a targeted manner to enhance cell density and stability, while aggregation and flocculation can facilitate cell harvest (153, 154). Moreover, cyanobacterial biofilms exhibit excellent heavy metal adsorption capabilities and are considered for wastewater treatment (155). Nitrogen-fixing species have potential use as natural fertilizers (156). In biophotovoltaics, T4P have been shown to enhance photocurrent generation due to tighter adherence and closer proximity of cells to the electrode surface (157). Recent findings indicate that indirect extracellular electron transfer via diffusible electron carriers is the mechanism facilitating photocurrent generation in *Synechocystis* (157–159), rather than direct electrical conductivity of T4P, as previously proposed (160, 161).

Cyanobacteria perform oxygenic photosynthesis, harnessing renewable sunlight and atmospheric carbon dioxide to generate carbohydrates and biomass. As climate change remains a pressing global challenge, these microorganisms offer promising potential for advancing sustainable, CO_2_-neutral industrial processes, serving as whole-cell catalysts in the biotransformation of valuable chemicals (reviewed in reference 162). Key to developing cyanobacteria as biotechnological chassis is the availability of reliable genetic manipulation tools (reviewed in reference 163). With regard to T4P, understanding the underlying mechanisms and regulation of natural competence could enhance the manipulation and exploitation of cyanobacteria. A practical example is the restoration of competence in *Synechococcus elongatus* UTEX 2973 by replacing a non-functional *pilN* with a functional copy from a related cyanobacterium (164). Conversely, T4P-mediated DNA uptake can be detrimental to industry-scale synthetic biology via unintended gene transfer (165).

## FUTURE DIRECTIONS

In summary, T4P-mediated behaviors in cyanobacteria are diverse and integral to their ecological success, facilitating adaptability and resilience in various habitats. Despite significant advances, several key questions remain unresolved in the field. One pressing challenge is to decipher the signaling pathway that translates light focusing by the cell body into polar pilus activity during phototaxis. Additionally, a more comprehensive understanding of how different minor pilins define the function of T4P is required. While T4P-mediated mechanosensing is well documented in heterotrophic bacteria (166), its presence and significance in cyanobacteria are not well understood. The potential for T4P to sense and respond to mechanical cues in the environment remains an open area of research. Another open question is how T4P contribute to flocculation and aggregation across various strains. Finally, unraveling the interplay between phototaxis and biofilm formation could provide insights into how cyanobacteria shape their natural multispecies co-habitats and influence the formation of complex, structured colonies. Addressing these gaps will advance our understanding of cyanobacterial behavior and its broader implications for both ecology and biotechnology.
